# Haplotype-resolved swine leukocyte antigen (SLA) gene typing using long-read whole genome sequencing

**DOI:** 10.3389/fimmu.2026.1903706

**Published:** 2026-10-16

**Authors:** Anne Boshove, Amalee E. Nunnally, Scott A. Callison, Jenelle R. Dunkelberger, Claudia A. Sevillano, Martien A. M. Groenen, Christine A. Jansen, Martijn F. L. Derks

**Affiliations:** 1 Animal Breeding and Genomics, Wageningen University & Research, Wageningen, Netherlands; 2 Topigs Norsvin Research Center, ‘s-Hertogenbosch, Netherlands; 3 Genomic Innovations Center, Ceva Animal Health, Shawnee, KS, United States; 4 Topigs Norsvin USA, Bloomington, MN, United States; 5 Cell Biology and Immunology, Wageningen University & Research, Wageningen, Netherlands

**Keywords:** immunogenetics, long-read sequencing, major histocompatibility complex (MHC), pig genomics, swine leukocyte antigen (SLA)

## Abstract

**Background:**

The Major Histocompatibility Complex (MHC) is a genomic region present in all vertebrates encoding genes essential for the immune response. In pigs, the MHC is named the Swine Leukocyte Antigen (SLA) complex. Traditional methods for SLA gene typing rely on targeted amplification and sequencing of SLA genes. In this study, we evaluated whether SLA genes can be identified from non-targeted Oxford Nanopore long-read sequencing data, to create a versatile approach that can be applied to existing long-read sequencing datasets.

**Methods:**

We analyzed 56 pigs from four breeds (Large White, Duroc, Landrace and a Large White-derived synthetic line) and generated *de novo* genome assemblies that were separated into haplotypes through phasing and haplotagging. SLA gene sequences from the IPD-MHC database were mapped to the haplotype-resolved assemblies, and candidate novel alleles and haplotypes were assessed using targeted NGS-based SLA typing.

**Results:**

This approach enabled confident allele assignment for over 95% of *SLA-1, SLA-2, SLA-3, SLA-DRB1*, and *SLA-DQB1* genes. The majority of results consisted of haplotypes which have been previously reported and additionally included several potential novel alleles and haplotypes. Targeted NGS-based SLA typing confirmed seven novel class I haplotypes as well as the presence of three novel *SLA-2* alleles, although additional sequencing is required to fully resolve the exon 2 sequence for two of them. We also observed strong linkage between assembly-derived SLA haplotypes and medium-density genotypes within the SLA region, supporting efficient identification and monitoring of SLA diversity in populations with similar linkage disequilibrium structures.

**Conclusion:**

Existing long-read sequencing and genotype datasets can be leveraged to characterize SLA variation, identify candidate novel haplotypes and alleles, and provide insight into SLA diversity within the pig populations studied.

## Introduction

The Major Histocompatibility Complex (MHC) is a genomic region present in all vertebrates, playing a central role in the immune system, particularly in adaptive immunity. The region is divided into three classes, of which the class I and class II regions encode the MHC-I and MHC-II molecules, respectively (1). These molecules bind antigens in a peptide-binding groove and are subsequently expressed on the surface of antigen-presenting cells, enabling T-cells to recognize presented antigens and initiate the cellular immune response. The peptide-binding groove of MHC-I consists of two alpha chains encoded by exons 2 and 3 of the MHC class I genes, whereas in MHC-II the groove is formed by an alpha- and beta-chain encoded by exon 2 of the corresponding class II genes. The amino acid composition and structure of this groove determine which peptides can be bound and presented on the cell surface. MHC genes are highly polymorphic, and variation in the peptide-binding regions can affect the repertoire of peptides that can be presented, thereby directly influencing the effectiveness of the immune response against different pathogens (2). This makes the MHC genes highly relevant to study on a genomic level.

In pigs, the MHC is referred to as the Swine Leukocyte Antigen (SLA) and is located on chromosome 7, spanning the centromere. The SLA class I region contains the three functional classical, highly polymorphic genes *SLA-1*, *-2* and *-3*, as well as several non-classical and pseudo-SLA genes. The class II region contains the *SLA-DQ*, *-DR*, *-DM* and *-DO* genes (3). To facilitate research into the MHC genes, the IPD-MHC database was established in 2003 (4). At the time of writing, it holds 100, 105, and 47 unique gene sequences for the *SLA-1, SLA-2* and *SLA-3* genes respectively. These gene sequences, or alleles as they are referred to in the database, can be identified by several methods such as polymerase chain reaction sequence-specific primers (PCR-SSP), PCR-restriction fragment length polymorphism (PCR-RFLP) (5, 6) and RT-PCR with cDNA sequencing (SBT) (7). More recently developed methods include genomic sequence-based high-resolution typing (GSBT) (8) and next generation sequence (NGS) based typing (9, 10). The information obtained through SLA typing is highly relevant for immune-related research. For example, it can enhance our understanding of how pigs respond to infectious diseases and vaccination (11), and can support epitope selection for peptide-based vaccine development (12).

In this study, we focus on four widely used pig populations: Large White, Landrace, Duroc, and a Large White-derived synthetic line. These breeds form the genetic backbone of modern pig production systems and are routinely genotyped using medium-density SNP arrays for genomic prediction in breeding programs. Such SNP-chips provide genome-wide marker information that is highly effective for estimating breeding values. Additionally, subsets of these populations are long-read whole genome sequenced (WGS) to perform more in-depth genomic analysis. Here, we explore the potential of using such routinely generated data for SLA typing. Constructing haplotype-separated assemblies from non-targeted Oxford Nanopore (ONT) long-read WGS data allowed us to create a reference framework of SLA haplotypes, which in-turn enabled the imputation of SLA alleles into large populations that are genotyped only with SNP arrays. We further used targeted NGS-based SLA typing on a separate dataset of 38 animals to validate candidate novel *SLA-2* alleles, and to assess whether we could infer their SLA haplotypes based on their SNP genotypes.

Together, this study presents complementary approaches for leveraging existing genomic data in SLA research, identifies several novel class I haplotypes and candidate *SLA-2* alleles, and provides insight into the distribution of SLA diversity within the pig breeds investigated.

## Materials and methods

### Dataset & long-read sequencing

The dataset consists of long-read sequence data and genotype data of 56 animals from five populations: four breeds (Large White, Landrace, Duroc, a Synthetic breed of Large White origin) and one cross (F1: Landrace x Large White). All animals were long-read sequenced using the ONT PromethION sequencer with either 9.4 or 10.4 chemistry (**Table 1**). The long-read sequence data was generated between 2022-2025, for which all blood samples were taken as part of routine sampling for breeding program purposes.

**Table 1:** Details and statistics for the Nanopore long-read sequencing of the study population.

Breed (Chemistry)	# Animals	# Sequenced on 1/2/3 flow cells	Avg. coverage 1 flow cell	Coverage 2/3 flow cells	Avg. read N50
Large White (9.4)	12	10/1/1	46	92/141	42
Duroc (9.4)	12	11/0/1	43	135	40
Synthetic (9.4)	4	3/0/1	48	150	40
Landrace (9.4)	16	15/0/1	47	140	42
Landrace x Large White (10.4)	12	12/0/0	36	–	32
Total	56	51	–	–	–

### Genome assembly

*De novo* genome assemblies of the four animals sequenced on three flow cells were previously constructed using Flye 2.9 (13) followed by polishing and scaffolding steps as described in (14). Genomes of the remaining 52 animals were *de novo* assembled using NextDenovo v2.5.2 (15) with a minimum read length of 3kb, a genome size of 2.7Gb and a seed depth of 40. All other parameters were set to default values.

### Resolving haplotypes

Each individual’s reads were mapped back onto their corresponding assemblies using minimap2 (16), with settings ‘-ax map-ont’ for Nanopore long reads and ‘—secondary=no’ to exclude secondary alignments. We used samtools v1.23 (17) to sort and index the resulting BAM files. All SLA allele sequences from the IPD-MHC database were mapped against the haploid genome assemblies using minimap2 (16). This allowed us to identify the contig(s) holding the SLA genes. We applied two approaches to obtain local haplotype-resolved assemblies. For the first approach, we extracted the contig(s) holding the SLA genes from both the individual’s BAM file and haploid assembly FASTA file. These files were given as input to HapDup 0.12 (13), which created two haplotype-resolved assemblies per individual. For the second approach, we performed variant calling on each individual’s BAM file using Clair3 v1.0.1 (18) where we used the r941_prom_sup_g5014 and r1041_e82_400bps_hac_v410 models for the samples sequenced on 9.4 and 10.4 chemistry respectively. Other settings used were ‘--platform=“ont”‘ to specify Nanopore data, ‘--include_all_ctgs’ to include all contigs of our assemblies and ‘--call_snp_only’ to exclude error-prone indels. This was followed by phasing and haplotagging using Whatshap 2.8 (19). The regions holding the SLA class I (850kb up- and downstream of *SLA-1*) and class II (200kb up- and downstream of *SLA-DQB1*) genes were extracted separately, and for each region the haplotagged reads were split into haplotype-separated FASTQ files using samtools. Each haplotype and region was assembled separately using Flye 2.9.6 (13).

### Allele mapping

The coding sequences of all SLA alleles from the IPD-MHC database (20) were mapped against each assembly using minimap2 with ‘-ax splice -uf’, enabling spliced alignment of exon sequences to genomic assemblies containing introns. Query sequences were assumed to be in forward transcript orientation (-uf) as IPD-MHC alleles are curated and strand-resolved. The ‘—MD’ flag was used to include the MD tag in the output. Exons 2 and 3 of *SLA-1, SLA-2* and *SLA-3*, and exon 2 of *SLA-DQB1* and *SLA-DRB1* encode the domains forming the peptide-binding groove, and therefore they are the minimal requirement for an allele to be included in the database. Hence, not all alleles have more than the sequences for exons 2 and 3 available, which is why we performed the mapping using these sequences. For each individual, we determined the alleles with the highest primary alignment score (AS) and lowest edit distance (NM). Additionally, we performed the mapping using the full gene coding sequences when available, of which the results were used to determine the allele for cases where several alleles share the same exon 2 and 3 sequences. Sam2pairwise (21) was used to obtain the pairwise alignment between the assembly and the alleles. Mapping results were used to identify and extract the regions holding the classical SLA Class I genes, underlying the self-alignment dot-plots which were created with NCBI BLAST (22) and the coverage/variant density plots which were created with matplotlib 3.10.3 (23).

### Genotyping & SNP haplotypes

All animals were genotyped using a custom Illumina Geneseek SNP-chip of 25K (27 animals) or 50K (29 animals) density. The genotypes were phased using Beagle 5.4 (24) together with a reference dataset of 20,000 animals genotyped at 50K density, while at the same time imputing the animals genotyped on the 25K SNP-chip to 50K density. A total of 55 SNPs were present in the SLA Class I region, which were filtered using PLINK 1.9 (25) on the non-imputed data using a minor allele frequency (MAF) threshold (--maf) of 0.1 and a per variant call rate threshold (--geno) of 0.1, leaving 29 SNPs after filtering. To reduce the total number of different SNP haplotypes, we excluded six SNPs that did not show any linkage to the SLA haplotypes. Finally, 23 SNPs were used to construct the Class I SNP haplotypes. Class II SNP haplotypes were constructed using nine SNPs from the 25K SNP-chip only, for which all animals had genotypes available (**Supplementary Table S1**). As these nine SNPs showed to be sufficient to distinguish between all Class II haplotypes present in the study population, we did not include any additional imputed SNPs from the 50K SNP-chip.

### Targeted NGS-based SLA typing

Thirty-two animals (Synthetic x Landrace x Large White) were typed using targeted Illumina sequencing of the SLA Class I region. RNA was extracted from samples using the ZymoBIOMICS DNA/RNA Miniprep Kit (Zymo Research) and treated with ezDNase Enzyme (Fisher Scientific) at 37 °C for 2 min. A denaturation step was performed using Extreme Thermostable Single-Stranded DNA Binding Protein (New England Biolabs) at 70 °C for 5 min prior to reverse transcription. cDNA synthesis was carried out using the Invitrogen SuperScript™ IV VILO™ Master Mix (Fisher Scientific) under the following conditions: 10 min at 25 °C, 60 min at 50 °C, and 5 min at 85 °C. cDNA libraries were prepared following the Illumina 16S metagenomic sequencing library preparation protocol (26) with SLA-specific primers substituted for 16S primers. SLA Class I primers were previously described (10) and were synthesized with Illumina adapter sequences appended to the 5′ ends and locked nucleic acids incorporated within the locus-specific regions to increase the melting temperature to approximately 62 °C. Libraries were sequenced on an Illumina MiSeq i100 Plus platform using a 5M-read reagent kit to generate 2 × 300 bp paired-end reads. Raw reads were first paired then merged using BBMerge (27). Merged reads were classified against the IPD-MHC SLA database (20) using CevaSEQ, a proprietary sequence analysis software platform developed by Ceva.

An additional six animals were sequenced following the same methods to validate the potential novel *SLA-2* alleles. Geneious Prime 2026.0.2 was used to find and extract sequences with identical residues. The twenty most prevalent sequences from each sample were then classified against the IPD-MHC Swine Leukocyte Antigen (SLA) database (4) using the IPD-MHC BLAST tool for allele classification. Any sequence that was found in high abundance that lacked an exact match in the IPD-MHC database was considered a candidate novel allele. Geneious Prime 2026.0.2 was used to visualize the multiple sequence alignments.

## Results

### *De novo* genome assembly and local haplotype-resolving

Our WGS dataset comprised Nanopore long-reads from 56 animals representing four breeds, which we used to construct haploid *de novo* genome assemblies for each individual. The assemblies yielded high contig N50 values, averaging between 64 and 70Mb for all purebred samples. The average contig N50 for the crossbred animals was notably lower at 34Mb, likely due to a combination of increased heterozygosity and lower read coverage and length.

To assess how the SLA region was assembled, we mapped the SLA allele sequences from the IPD-MHC database to the haploid assemblies and evaluated the number of different contigs to which the alleles aligned. In 12 samples, all alleles mapped to a single contig, indicating that the entire SLA region was assembled contiguously. In most samples, however, Class I and Class II alleles mapped to different contigs, likely reflecting the difficulty of resolving the centromere which separates these classes. In two samples we observed alleles mapping to a total of three different contigs. In these cases, the third contig only contained mappings of SLA pseudogenes.

While this initial mapping was able to identify the locations of the SLA genes, the mapping quality was suboptimal as the assemblies contained a mixture of both haplotypes. Accurate typing of SLA alleles therefore required haplotype-resolved assemblies. For each individual, the contigs holding the SLA genes were extracted and separated into haplotypes using HapDup. While this approach performed well overall, we observed 14 samples in which haplotypes remained partially collapsed. In these regions we observed long stretches without any called variants, which prevented haplotype separation. To resolve this, we applied a second approach using a different variant caller, followed by reassembly of the SLA region using haplotype-separated reads. Although these assemblies were more fragmented and failed to properly reconstruct the SLA region for a small number of individuals, this approach successfully resolved the haplotypes that HapDup kept collapsed.

### SLA allele typing of haplotype-resolved assemblies

To identify the SLA alleles carried by each individual, we mapped the sequences of all SLA alleles listed in the IPD-MHC database against the constructed haplotype-separated assemblies and determined the best-matching allele for *SLA-1, SLA-2, SLA-3, SLA-DRB1* and *SLA-DQB1*. Allele assignments were classified based on concordance between the exon 2 and 3 sequences of the assembly and the database allele with highest mapping scores (**Table 2**). When either assembly method returned an allele alignment with 100% identity, we considered these confident matches. In several cases, the alignment contained one or two single base pair insertions or deletions. Such inconsistencies can arise either through sequencing errors, inaccuracies in assembly or variant calling or incomplete phasing. Additionally, such indels would lead to a frameshift and render a non-functional protein. Therefore, we treated these indels as method-specific errors and corrected them, counting these alleles as indel-corrected matches. Two cases carried 5–6 insertions, which were both treated as low-confidence matches.

**Table 2:** Confidence of SLA allele assignments across five genes based on long-read whole-genome sequencing.

Gene	Confident match	Indel corrected match	Potential novel allele	Low confidence match	Unresolved
*SLA-1*	113	1	0	3	3
*SLA-2*	98	3	8	3	1
*SLA-3*	92	12	0	7	1
*SLA-DRB1*	106	3	0	3	0
*SLA-DQB1*	93	19	0	0	0

When SNP mismatches occurred between the assembly and the database allele, we considered whether the same mismatches appeared in other samples carrying the same allele, as recurring differences could indicate a novel allele. Alleles with SNP mismatches unique to a single animal and gene were considered low confidence if there were three or fewer base pair differences, or unresolved if there were four or more. We identified eight cases of *SLA-1* gene duplication, resulting in a total of 120 *SLA-1* alleles. Overall, ~95% of all genes perfectly matched with an SLA allele from the database after indel correction, demonstrating that our long-read assembly-based approach enables accurate SLA gene identification.

### Identifying novel alleles for SLA-2

We identified two potential novel alleles for *SLA-2* in our population. Three animals carried the same five base pair mismatches relative to SLA-2*07:03, all located in exon 2. Their exon 3 sequence is identical to the exon 3 sequence of SLA-2*07:03. All five mismatches lead to a change in amino acid. Four of the amino acid changes in exon 2 relative to allele 07:03 are found in the sequences of other 07:* alleles. Only one of the changed amino acids is not found in any other 07:* alleles, but is found in alleles 02:* and 04:*. Another five animals shared the same four mismatches relative to SLA-2*06:01, all located in exon 3. Their exon 2 sequence is identical to the exon 2 sequences of SLA-2*06:01, SLA-2*06:07 and SLA-2*06:16. The mismatches lead to two changes in amino acids, both found in other 06:* alleles (**Table 3**).

**Table 3:** Amino acid (AA) changes found in three animals relative to allele SLA-2*07:03 and five animals relative to SLA-2*06:01.

Closest allele	AA position	AA in candidate novel allele	Found in
SLA-2*07:03	30	R	07:05
	33	D	02:*, 04:*
	101	S	07:01, 07:02, 07:06
	103	N	07:01, 07:02, 07:06
	104	N	07:01, 07:02, 07:06
SLA-2*06:01	176	E	06:02, 06:03, 06:05, 06:08, 06:10
	179	G	06:11, 06:12, 06:13

The presence of these amino acids at the same positions in other alleles supports the biological plausibility of these candidate novel alleles and indicates that they could have arisen through recombination between existing *SLA-2* alleles.

To validate these candidate novel *SLA-2* alleles identified from the long-read assemblies, targeted sequencing of *SLA-1*, *SLA-2* and *SLA-3* was performed for three animals per candidate novel allele. For each candidate allele, we generated multiple sequence alignments including the closest matching IPD-MHC allele as reference, the sequence inferred from the long-read assemblies, and the sequences obtained through targeted sequencing. For the candidate novel allele resembling SLA-2*06:01, the targeted sequencing results were fully concordant with the sequence obtained from the long-read sequencing-based assemblies (**Figure 1**).

**Figure 1:**
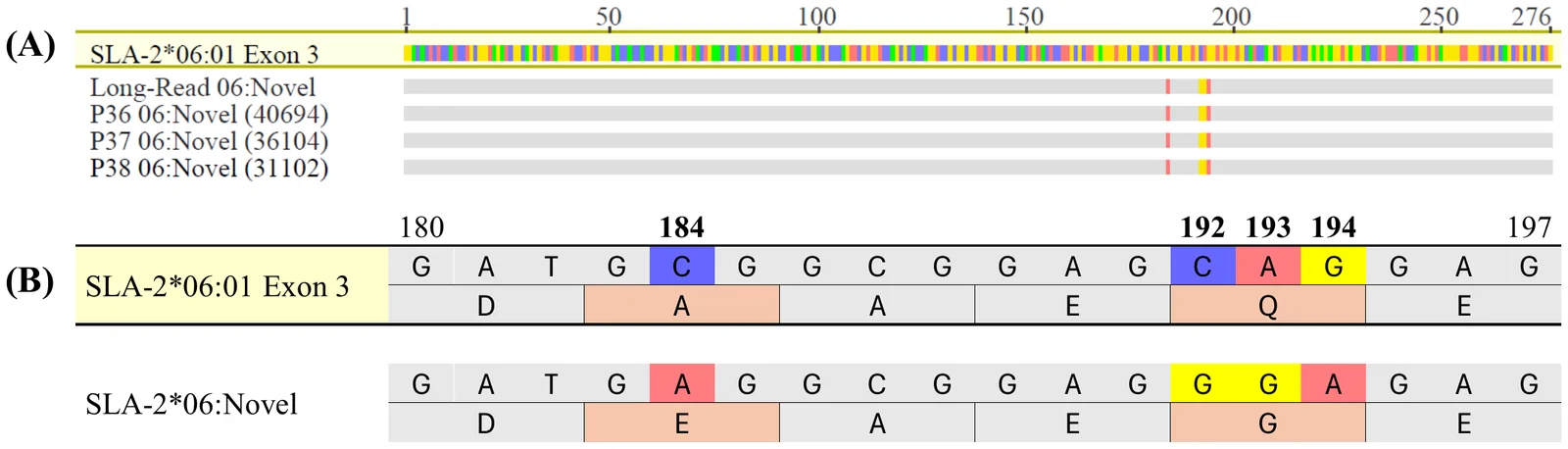
**(A)** Multiple sequence alignment of exon 3 of IPD-MHC database allele SLA-2*06:01 with the corresponding candidate novel allele sequence identified from long-read sequencing-based assemblies and sequences obtained by targeted sequencing of individuals carrying the candidate novel allele; targeted sequencing read counts are shown in brackets. **(B)** Zoomed-in view showing nucleotide and amino acid differences between SLA-2*06:01 exon 3 and the novel allele sequence.

For the candidate novel allele resembling SLA-2*07:03, two of the nucleotide differences detected in the long-read assembly were located within the first 87 base pairs of exon 2, a region not covered by the targeted sequencing assay. The other three nucleotide differences were fully concordant between all sequences (**Figure 2**). Therefore, the targeted sequencing data confirms the presence of a novel allele, but does not yet resolve the complete exon 2 sequence. Additional sequencing covering the full exon 2 will be required to verify the complete allele sequence.

**Figure 2:**
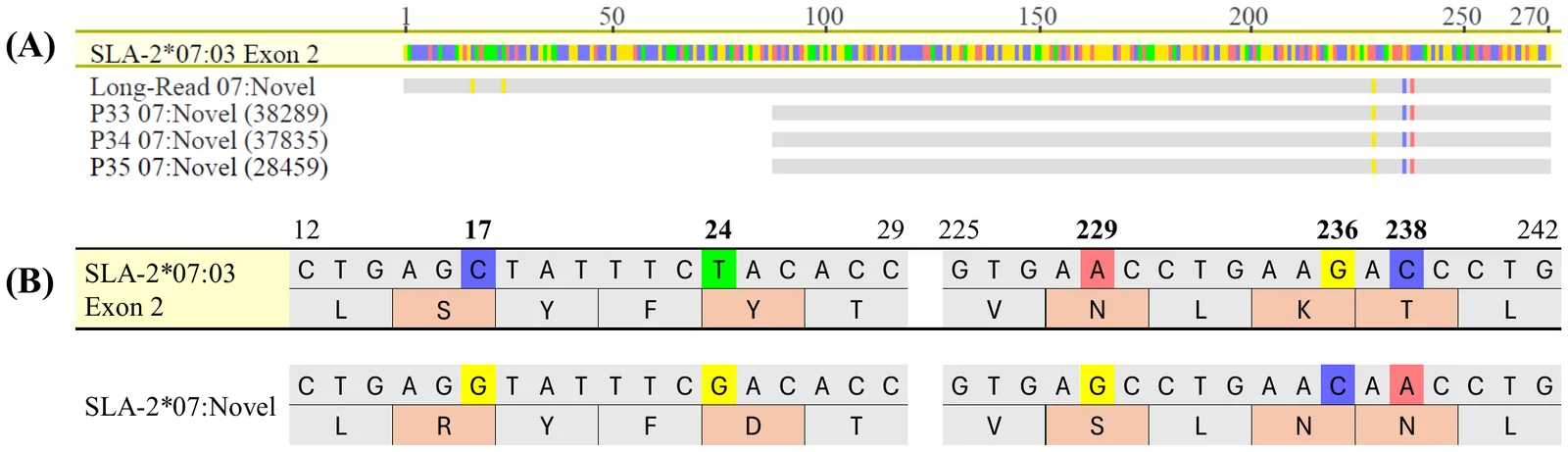
**(A)** Multiple sequence alignment of exon 2 of IPD-MHC database allele SLA-2*07:03 with the corresponding candidate novel allele sequence identified from long-read sequencing-based assemblies and sequences obtained by targeted sequencing of individuals carrying the candidate novel allele; targeted sequencing read counts are shown in brackets. **(B)** Zoomed-in view showing nucleotide and amino acid differences between SLA-2*07:03 exon 2 and the novel allele sequence.

### Identifying known and potential novel SLA haplotypes

The set of linked SLA alleles co-inherited constitutes an SLA haplotype. Many haplotypes have been previously identified and described in literature, of which most have received an official name designation. Class I haplotypes include *SLA-1, SLA-2, SLA-3* and can additionally include *SLA-6*. Class II haplotypes include *SLA-DRB1, SLA-DQB1*, and can additionally include *SLA-DQA* and *SLA-DRA*. Class I and Class II can be considered together as one haplotype, however, as we could not confidently resolve parental phase across both classes, we considered them separately.

#### Phasing & copy number variation

When comparing the haplotype results to known haplotypes from literature as well as to the results of other animals within the population, we identified six cases where we suspected phasing errors between *SLA-1* and *SLA-3.* In these cases, the animals appeared to carry known haplotypes, but with the *SLA-1* alleles exchanged between haplotypes. The raw, computationally phased haplotypes alongside our reconstructions based on prior knowledge are described in **Supplementary Table S2**.

These apparent phase switches may be explained by copy number variation (CNV) of SLA genes and pseudogenes, which can complicate read mapping, variant calling and phasing. To obtain an approximate indication of SLA gene copy number present in the assemblies, we performed self-alignments and identified regions with repeated alignments, as SLA genes are expected to align to one another due to their high sequence similarity. In addition, we inspected read depth after mapping the reads back to the assemblies, together with the density of called variants across the region.

Two of the haplotypes involved in the suspected phase switches were expected to correspond to Hp-28 (samples 3 and 6), a previously described haplotype containing an *SLA-1* duplication and several duplicated SLA pseudogenes, resulting in a total of 11 SLA gene or pseudogene copies (28) from *SLA-1* up to and including *SLA-2*. Self-alignment of the assemblies of samples 3 and 6 indicated the presence of eight gene copies (**Figure 3**; **Supplementary Figure S5**), suggesting that part of the expected duplicated structure was represented in the assemblies.

**Figure 3:**
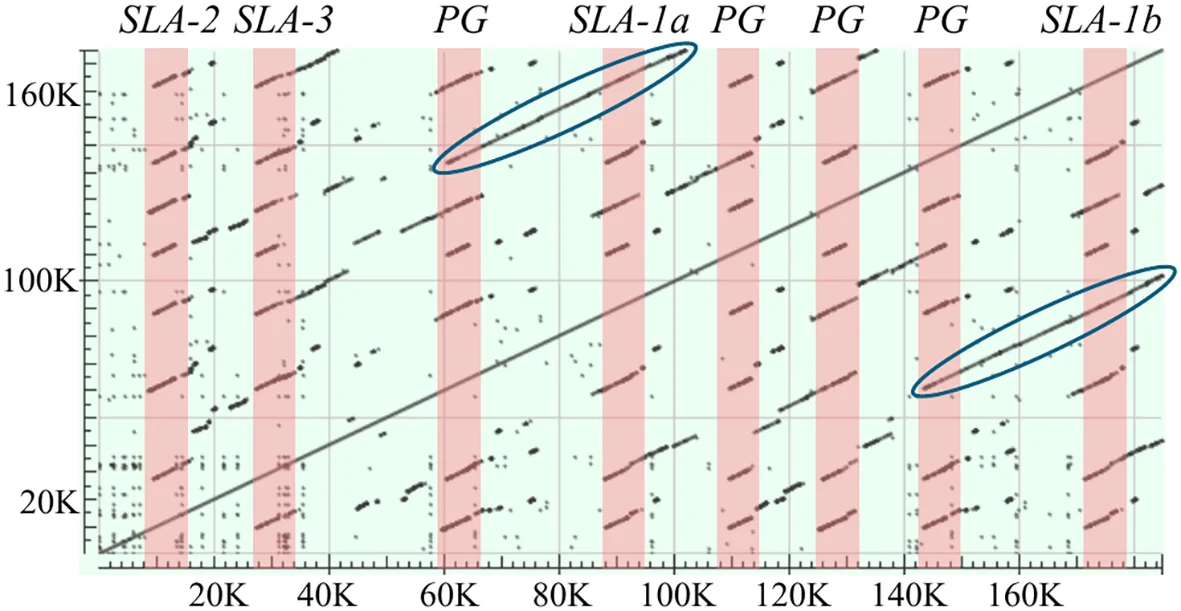
Dot-plot showing a self-alignment of sample 3 for the region spanning the classical SLA Class I genes, with all SLA gene copies showing up as repeat regions. SLA gene locations are highlighted in red. Circled in blue is a duplication resulting in an additional copy of SLA-1. PG = Pseudogene.

Inspection of read depth and variant density across this region revealed a large reduction in coverage and absence of called variants in both samples, located between the two *SLA-1* copies (**Figure 4**; **Supplementary Figure S5**). This could represent a duplicated segment that is present on one haplotype, likely Hp-28, but absent from the other. As a result, no heterozygous variants were called across this interval, preventing reliable phasing through the region and providing a plausible explanation for the observed phase switches. Both samples also showed local increases in coverage, which may reflect the additional Hp-28 gene copies that were not resolved in the assemblies.

**Figure 4:**
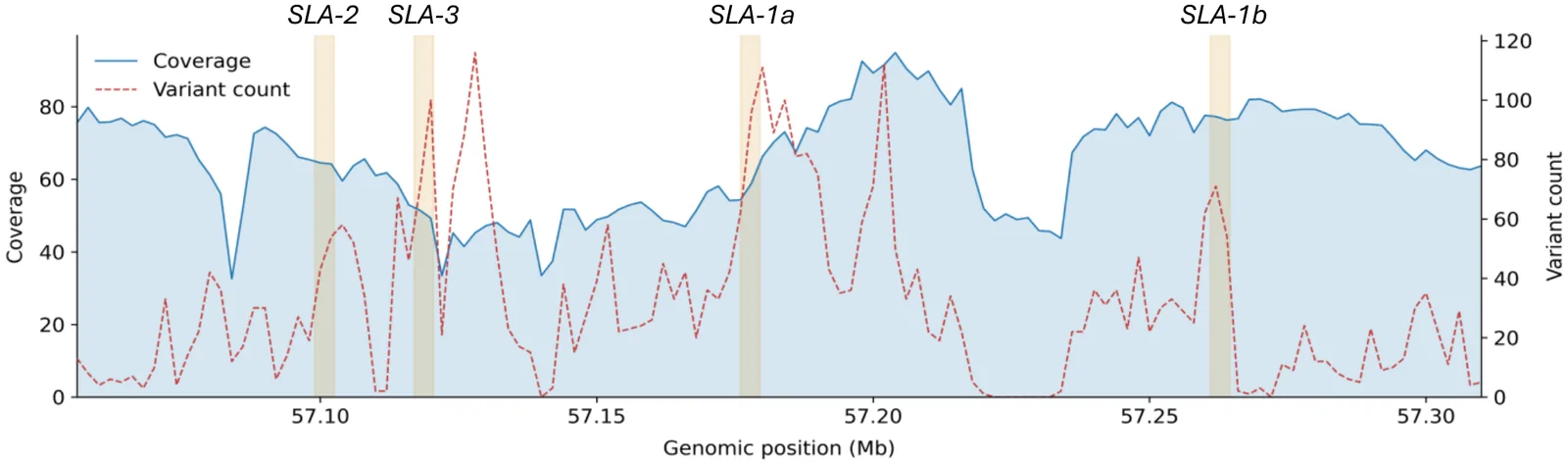
Self-mapped read coverage and heterozygous variant density across the region holding the classical SLA Class I genes of sample 3. Mean coverage (blue) and the number of detected variants (red, dashed) per 2000bp genomic window are shown across the region. Highlighted regions indicate the approximate locations of SLA genes, generally showing higher variant density.

Similar patterns were observed in the remaining samples with suspected phase switches, although the extent of the evidence varied between individuals. Sample 1 contained three additional assembled gene copies and showed two extended regions without called variants, together with coverage peaks suggestive of additional gene copies that were not resolved in the assembly (**Supplementary Figure S1**). Samples 4 and 5 also showed additional assembled gene copies, ranging from three to six, as well as coverage patterns consistent with further unresolved copies and regions of reduced variant density (**Supplementary Figures S3, S4**). In sample 2, no additional gene copies were detected in the assembly; however, the local increase in coverage suggests that copy number variation may still have been present but incompletely represented in the assembled sequence (**Supplementary Figure S2**). Taken together, the fact that the reconstructed haplotypes correspond to previously described haplotypes, and the presence of CNV-associated phasing limitations support the interpretation that these cases represent phasing errors rather than true independent recombination events. We therefore considered the reconstructed haplotypes to be the more biologically plausible combinations and used these haplotypes in subsequent analyses.

#### Class I haplotypes

In our analysis, four Class I haplotypes were excluded as they contained unresolved alleles. The remaining 108 made up 22 distinct haplotypes, of which 16 have been previously described in literature (**Table 4**; **Figure 5**) (20, 29, 30). We identified six potential novel class I haplotypes, including two (New^1^ and New^2^) that carry the novel *SLA-2* alleles, which are referred to as 07:Novel and 06:Novel. Though not present in this study population, haplotype New^1^ could have emerged as a recombinant of Hp-91.0, which carries the same alleles for *SLA-1* and *SLA-3* but carries *SLA-2* allele 07:01 (31). Haplotype New^2^ seems to be most similar to known haplotype 37.0, which also carries alleles 07:04 and 05:06 for *SLA-1* and *SLA-3* respectively, but carries allele 09:05 for *SLA-2*. The *SLA-1* duplication on haplotype New^3^ has been described previously (8), but has to our knowledge not been assigned to a full haplotype. Haplotypes New^4^, New^5^ and New^6^ could all be results of recombination events between haplotypes present in this population. Haplotype New^5^, however, is also highly similar to haplotype 46.0, with the only difference being that haplotype 46.0 carries only a single copy of *SLA-1*, which is allele 12:01.

**Table 4:** SLA Class I haplotypes (Hp) identified in Duroc, Synthetic (SY), Large White (LW), Landrace (LR), and Landrace x Large White crossbreds (LRxLW).

Hp	*SLA-1*	*SLA-3*	*SLA-2*	Count	Duroc	SY	LW	LR	LRxLW
1.0	01:01	01:01	01:01	11	–	–	9	–	2
4a.0	04:01	04:01	04:01	11	2	3	5	–	1
4b.0	04:01	04:01	04:02	8	8	–	–	–	–
5.0	04:02	05:03	08:01	2	–	–	1	–	1
7.0	08:01	07:01	05:02	1	–	–	–	–	1
25.0	11:01	03:02	07:01	7	–	–	–	4	3
26a.0	08:08	05:02	10:05	1	–	1	–	–	–
26b.0	08:11	05:02	10:06	3	–	–	–	1	2
28.0	15:01 + 09:01	07:01	05:03	4	–	–	–	1	2
32.0	07:02	04:02	02:02	10	7	–	–	2	1
35.0	12:01 + 13:01	05:02	10:01	1	–	–	1	–	–
43.0	11:04	04:01	04:02	2	2	–	–	–	–
55.0	14:02	04:03	11:04	25	–	4	5	14	2
56.0	11:02	03:03	15:01	3	3	–	–	–	–
62.0	14:01	04:04	06:02	2	–	–	–	2	–
73.0	15:01	04:02	02:02	2	–	–	–	2	–
New^1^	15:03	03:02	07:Novel	3	–	–	–	2	1
New^2^	07:04	05:06	06:Novel	5	–	–	3	–	2
New^3^	21:01 + 11:03	05:03	16:03	2	–	–	–	–	2
New^4^	07:02	04:01	04:01	1	1	–	–	–	–
New^5^	12:01 + 13:01	04:04	06:02	1	–	–	–	1	–
New^6^	08:01	04:02	02:02	3	–	–	–	1	2

**Figure 5:**
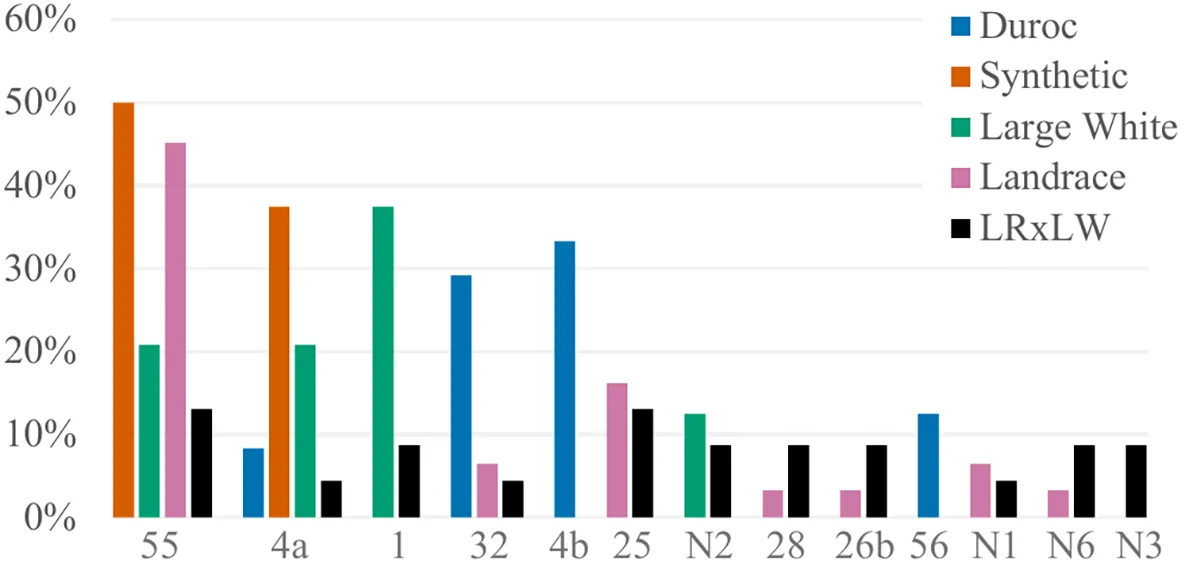
Clustered bar chart with X-axis showing all SLA Class I haplotypes that occurred three times or more in the study population and Y-axis showing the percentage at which the haplotype occurred in each breed.

Out of the previously identified haplotypes, Hp-55.0 is by far the most frequently occurring in this population. It was first described in the ESK-4 cell line (32), and later found to be the seemingly singular haplotype carried by the highly inbred Babraham pigs, which are derived from a Large White background (33). While we observe the haplotype in Large White, the highest frequency is found in Landrace. The other most abundant haplotypes found were Hp-1.0, Hp-4a.0, Hp-4b.0, Hp-25.0 and Hp-32.0, which have all been previously reported in similar pig populations (29, 30, 34).

#### Class II haplotypes

We identified 19 distinct Class II haplotypes (**Table 5**; **Figure 6**) (20, 29, 31). Here we did not find a single high-frequent haplotype, as with Class I. Instead, we found the three most frequent haplotypes, Hp-0.2, Hp-0.6 and Hp-0.12b, to each occur 14 times. Hp-0.2 was found exclusively in Duroc, and Hp-0.6 and Hp-0.12b were more frequent in Landrace, which is in line with results from previous studies within these breeds (34). The haplotype listed as ‘NA’ has been previously described, but we were unable to find an official name designation (35).

**Table 5:** SLA Class II haplotypes (Hp) identified in Duroc, Synthetic (SY), Large White (LW), Landrace (LR), and Landrace x Large White crossbreds (LRxLW).

Hp	*SLA-DRB1*	*SLA-DQB1*	Count	Duroc	SY	LW	LR	LRxLW
0.1	01:01	01:01	12	–	–	10	–	2
0.2	02:01	02:01	14	14	–	–	–	–
0.4	02:01	04:01	3	–	–	2	–	1
0.5	05:01	02:01	2	2	–	–	–	–
0.6	05:01	08:01	14	–	–	–	11	3
0.10	04:01	08:01	1	1	–	–	–	–
0.11	09:01	04:02	2	2	–	–	–	–
0.12a	06:02	07:01	5	–	–	–	5	–
0.12b	06:01	07:01	14	–	–	3	7	4
0.14	09:01	08:01	9	2	3	1	–	3
0.15b	04:02	02:02	4	–	4	–	–	–
0.20	06:02	03:03	1	–	1	–	–	–
0.23	10:01	06:01	8	–	–	1	3	4
0.25	13:01	09:01	7	–	–	–	4	3
0.26	11:02	04:03	4	–	–	2	–	2
0.27	09:01	09:01	4	–	–	4	–	–
0.30	11:01	05:03	3	3	–	–	–	–
0.41	04:01	05:01	3	–	–	–	2	1
NA^1^	05:02	06:01	2	–	–	1	–	1

^1^ Haplotype previously described but without name designation.

**Figure 6:**
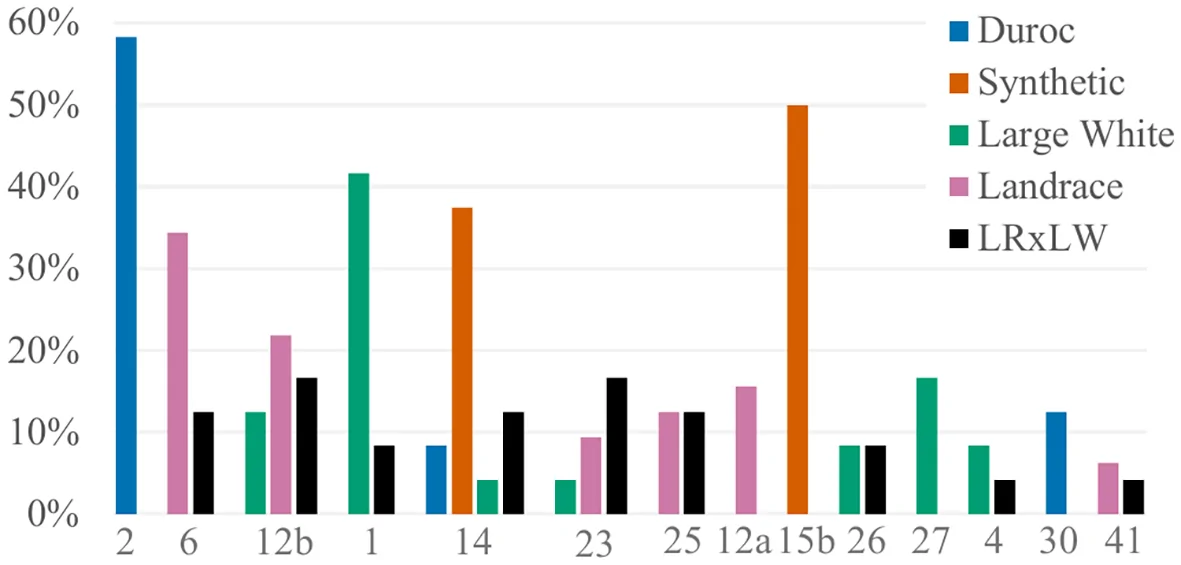
Clustered bar chart with X-axis showing all SLA Class II haplotypes that occurred three times or more in the study population and Y-axis showing the percentage at which the haplotype occurred in each breed.

#### SNP-based haplotypes

Having established SLA allele and haplotype assignments from the long-read assemblies, we next assessed the potential of using routinely available medium-density genotype data to investigate SLA diversity. To this end, we constructed SNP-based haplotypes for each animal using 23 SNPs distributed across the class I region and 9 SNPs across the class II region, which were in moderate to low linkage disequilibrium (**Supplementary Figures S6–9**). We evaluated the co-occurrence of SNP haplotypes with SLA haplotypes and observed a strong linkage. For Class I, 17 SLA haplotypes occurred exclusively with a single unique SNP haplotype, while five SLA haplotypes occurred with multiple SNP haplotypes that too were unique to the respective SLA haplotypes. In this dataset, only SLA haplotypes 32.0 and 73.0 could not be distinguished as they shared the same SNP haplotype (**Figure 7A**). For class II, 15 SLA haplotypes occurred with a single unique SNP haplotype, four SLA haplotypes occurred with multiple unique SNP haplotypes, and none of the SLA haplotypes shared the same SNP haplotype (**Figure 7B**). These results are consistent with the phase-corrected haplotype reconstructions and further indicate that the six potential novel haplotypes are distinguishable from the other haplotypes observed in this dataset.

**Figure 7:**
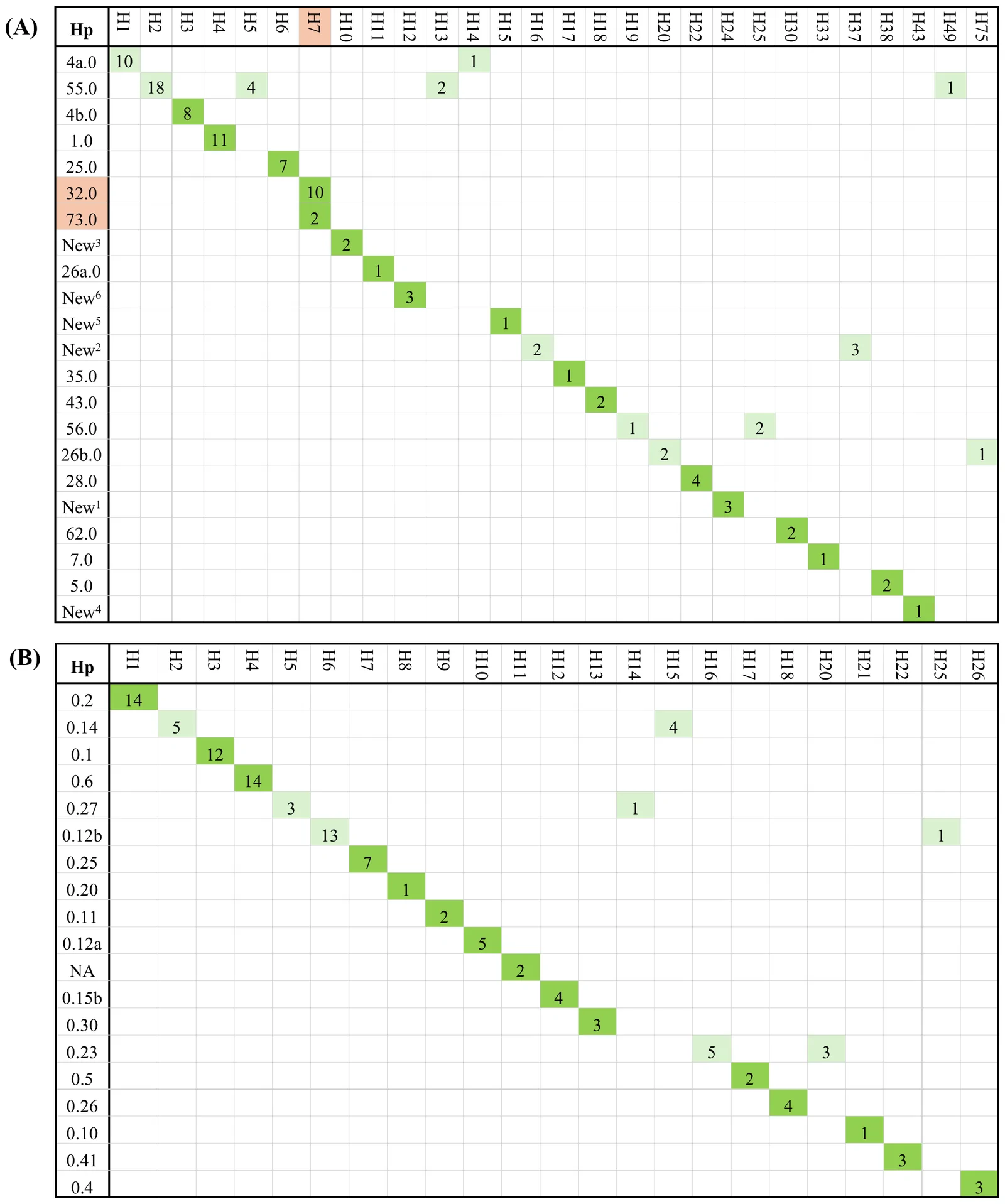
Co-occurrence of SLA haplotypes (Y-axis) and SNP-based haplotypes (X-axis) for **(A)** Class I and **(B)** Class II. Cell values indicate the number of animals carrying a given combination. SNP-haplotype names are unique for each class and do not refer to the same haplotype.

As we are able to confidently phase the genotypes, these results allowed us to determine which Class I and Class II SLA haplotypes belonged to the same parental haplotype. We found the linkage between Class I and Class II to be relatively low, with a total of 53 distinct haplotypes (**Supplementary Table S3**). Thirteen haplotypes occurred more than twice, with the most common haplotype, Hp-55.6, occurring nine times. Notably, this is the exact haplotype as found in the highly inbred Babraham pigs (33).

### Validating SNP-based SLA inference with targeted NGS-based typing results

To further assess and verify the inference of SLA haplotypes based on SNP haplotypes, we performed an additional analysis on a separate dataset. This dataset consisted of 32 F2 animals (Synthetic x Landrace x Large White) for which both SNP-chip results and targeted NGS-based SLA Class I typing results were available. Additionally, the table includes the six animals that were targeted to sequence the two candidate novel *SLA-2* alleles (P33-P38). This allowed us to construct a total of 76 SNP haplotypes, of which 54 were present in our long-read sequenced population (**Table 6**). Each inference made based on SNP haplotype corresponds with what is observed through targeted NGS-based typing. SNP haplotype H35 corresponds to an SLA haplotype of 08:10, 12:01, 06:01, which has been previously described as Hp-22 (34).

**Table 6:** SNP-based haplotypes (SNP HP) and their linkage-inferred SLA Class I haplotype (Hp) for 38 animals, combined with results from NGS-based typing.

Sample(s)	SNP HP/Hp (1)	SNP HP/Hp (2)	*SLA-1* typing	*SLA-2* typing	*SLA-3* typing
P1, P2	H1/Hp-4a	H2/Hp-55	04:01, 14:02	04:01, 11:XX	04:XX, 04:XX
P3	H1/Hp-4a	H13/Hp-55	04:01, 14:02	04:01, 11:XX	04:XX, 04:XX
P4	H1/Hp-4a	H3/Hp-4b	04:01	04:01, 04:02	04:XX
P5, P6	H1/Hp-4a	H6/Hp-25	04:01, 11:01	04:01, 07:01	04:XX, 03:02
P7, P8	H1/Hp-4a	H9/NA	04:01, 21:03	04:01, 06:04	04:05, 04:XX
P9	H1/Hp-4a	H137/NA	04:01, 21:03	04:01, 06:04	04:05, 04:XX
P10	H1/Hp-4a	H21/NA	04:01, 14:01	04:01, 06:02	04:04, 04:XX
P11	H2/Hp-55	H95/NA	07:02, 14:02	02:02, 11:X	04:XX, 04:XX
P12	H2/Hp-55	H10/New^3^	11:03, 14:02 21:01	11:XX, 16:03	04:XX, 05:03
P13	H3/Hp-4b	H7/Hp-32 or Hp-73	04:01, 15:01	02:02, 04:02	04:XX, 04:XX
P14	H3/Hp-4b	H12/New^6^	04:01, 08:01	02:02, 04:02	04:XX, 04:XX
P15	H3/Hp-4b	H20/Hp-26b	04:01, 08:11	04:02, 10:06	04:XX, 05:02
P16	H3/Hp-4b	H16/New^2^	04:01, 07:04	04:02, 06:Novel	04:XX, 05:07
P17	H4/Hp-1	H45/NA	01:01, 14:02	01:01, 11:XX	01:01, 04:XX
P18	H5/Hp-55	H30/Hp-62	14:01, 14:02	06:02, 11:XX	04:04, 04:XX
P19	H5/Hp-55	H21/NA	14:01, 14:02	06:02, 11:XX	04:04, 04:XX
P20	H6/Hp-25	H23/NA	11:01, 14:02	07:01, 11:XX	03:02, 04:XX
P21	H7/Hp-32 or Hp-73	H10/New^3^	07:02, 11:03, 21:01	02:02, 16:03	04:XX, 05:03
P22	H7/Hp-32 or Hp-73	H20/Hp-26b	07:02, 08:11	02:02, 10:06	04:XX, 05:02
P23	H7/Hp-32 or Hp-73	H51/NA	07:02, 21:03	02:02, 06:04	04:05, 04:XX
P24	H38/Hp-5	H97/NA	04:02, 14:02	08:01, 11:XX	04:XX, 05:03
P25, P26	H8/NA	H4/Hp-1	01:01, 08:17	01:01	01:01, 07:01
P27	H8/NA	H7/Hp-32 or Hp-73	07:02, 08:17	02:02	04:XX, 07:01
P28	H8/NA	H9/NA	21:03, 08:17	06:04	04:05, 07:01
P29	H8/NA	H13/Hp-55	14:02, 08:17	11:XX	04:XX, 07:01
P30	H8/NA	H35/NA	08:10, 08:17	12:01	06:01, 07:01
P31	H1/Hp-4a	H28/NA	04:01, 07:02	04:01	04:XX, 07:01
P32	H4/Hp-1	H28/NA	01:01, 07:02	01:01	01:01. 07:01
P33, P35	H24/New^1^	H78/NA	07:02, 15:03	04:XX, 03:02	02:02, 07:Novel
P34	H24/New^1^	H3/Hp-4b	04:01, 15:03	04:XX, 03:02	04:02, 07:Novel
P36	H16/New^2^	H3/Hp-4b	04:01, 07:04	04:XX, 05:07	04:02, 06:Novel
P37, P38	H37/New^2^	H3/Hp-4b	04:01, 07:04	04:XX, 05:07	04:02, 06:Novel

NA indicates this SNP haplotype was not present in the long-read sequenced population and therefore no inference could be made.

#### Identification of additional novel SLA-2 allele

The SLA haplotype linked to SNP haplotype H8 appears to carry a novel allele for *SLA-2*, as only a single allele was detected in samples P25-P30. Our long-read sequenced population included one animal with SNP haplotype H8, which wasn’t included in further analysis due to the *SLA-2* allele being unresolved. The SLA haplotype linked to SNP haplotype H28 is also suspected to carry a novel allele for *SLA-2.* Closer inspection of the targeted sequencing results for the samples carrying these two haplotypes (P25-32) revealed a single novel *SLA-2* allele shared by both haplotypes (**Figure 8A**). This novel allele showed 99% identity to allele SLA-2*06:12, with seven base-pair mismatches resulting in two amino acid changes. When additionally including the sequence from the single long-read sample carrying this novel allele, we find the mismatches are concordant with the novel allele identified through targeted sequencing. However, the long-read sequence contained two additional base pair mismatches at the start of exon 2, resulting in two additional amino acid changes (**Figures 8B, C**). Targeted sequencing covering the full exon 2 sequencing will therefore be required to confirm the complete sequence of this novel allele.

**Figure 8:**
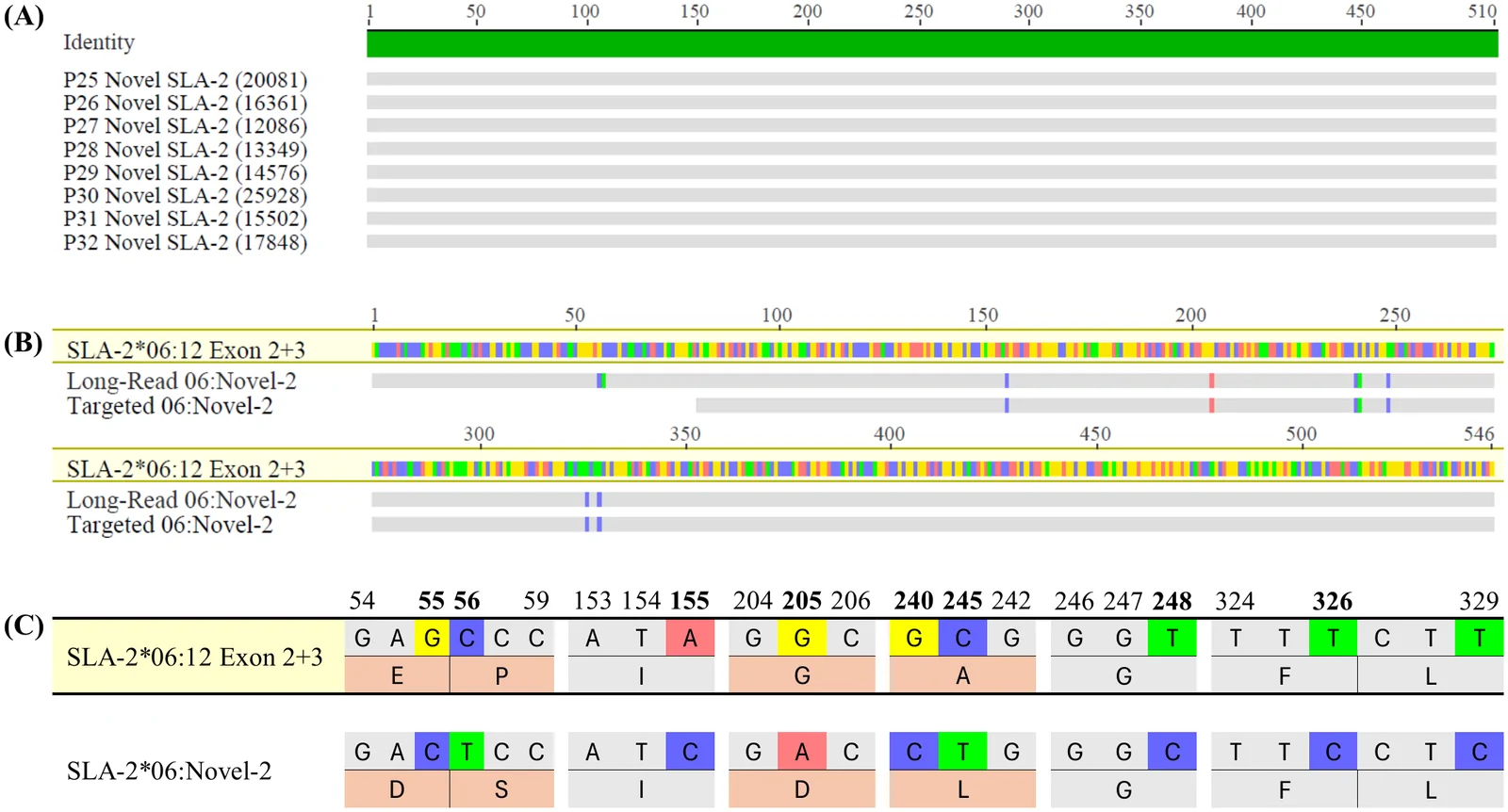
**(A)** Multiple sequence alignment of the novel SLA-2 sequence identified in eight samples through targeted sequencing, with read counts in brackets. **(B)** Multiple sequence alignment of exon 2 and 3 of IPC-MHC database allele SLA-2*06:12 with the corresponding novel allele sequence identified from a long-read sequencing-based assembly and the sequence obtained by targeted sequencing. **(C)** Zoomed-in view showing nucleotide and amino acid differences between SLA-2*06:12 exons 2 and 3 and the novel allele sequence.

#### Overview of novel haplotypes

We can derive that SNP haplotypes H9, H51 and H137 correspond to an SLA haplotype consisting of alleles SLA-1*21:03/SLA-2*06:04/SLA-3*04:XX, which is a combination that, to our knowledge, has not previously been described in literature. Additionally, the targeted sequencing results revealed two novel haplotypes sharing a novel *SLA-2* allele, and confirmed novel haplotypes New^1^, New^2^, New^3^ and New^6^ which were initially identified through long-read sequencing. Together, we identified a total of seven novel class I haplotypes that have been confirmed through targeted sequencing, including three novel allele sequences for *SLA-2* (**Table 7**).

**Table 7:** Overview of all SLA Class I haplotypes identified in this study that to our knowledge have not been described before, and the frequencies at which they were observed in our long-read sequence and targeted sequence datasets.

ID	*SLA-1*	*SLA-3*	*SLA-2*	#Long-read sequenced	#Targeted sequenced
New^1^	15:03	03:02	07:Novel	3	3
New^2^	07:04	05:06	06:Novel	5	4
New^3^	21:01 + 11:03	05:03	16:03	3	2
New^6^	08:01	04:02	02:02	3	1
New^7^	07:02	07:01	06:Novel-2	0	2
New^8^	08:17	07:01	06:Novel-2	1	6
New^9^	21:03	04:05	06:04	0	5

## Discussion

In this study, we explored the feasibility of leveraging existing genomic datasets to characterize SLA alleles and haplotypes. By aligning known SLA allele sequences from the IPD-MHC database to local haplotype-resolved assemblies constructed with Oxford Nanopore long-read WGS data, we were able to identify SLA alleles and haplotypes for the majority of animals in a cohort consisting of four pig breeds. By linking these assembly-derived SLA haplotypes to medium-density SNP-chip genotypes, we further evaluated whether SLA haplotypes could be inferred from routinely available genotype data. Together, these results show that long-read WGS data can support detailed SLA allele and haplotype discovery, while linked SNP haplotypes may provide a scalable approach for SLA haplotype inference within populations with similar linkage disequilibrium structures.

The approach presented here offers the potential to leverage existing long-read WGS datasets, enabling large-scale investigation into SLA diversity across various populations. Naturally, the availability and quality of the sequencing data can influence the accuracy of this type of analysis. An essential step is accurate phasing of the data to separate the haplotypes. One of the most reliable methods to do so is through trio-binning, which phases on read level before assembly and involves assigning each read to a parent using sequence data from both parents (36). Through this method it can also reliably be determined which haplotype an animal inherited from which parent. Alternative methods for haplotype-resolved assembling without the need of parental data have been based on the use of Hi-C data (37) and strand-sequencing (38). Constructing accurate haplotype-resolved assemblies from merely Nanopore data, however, remains a non-trivial task. We opted to generate haploid assemblies for the entire genome and attempt to resolve the haplotypes for only the contigs containing the SLA region. We used NextDenovo to generate the initial assemblies, as this tool is able to produce contiguous assemblies in which the SLA classes were assembled onto single contigs, easing further analysis. We proceeded to apply two approaches to separate the haplotypes, one using HapDup and one using Clair3 and Flye. The use of two independent pipelines relying on different tools can help in identifying and correcting method-specific errors and increase confidence in allele assignments where the results are concordant. HapDup performed best overall, with the main drawback being that its variant caller PEPPER (39) failed variant calling in certain regions which resulted in locally collapsed haplotypes. Clair3 proved to be a more suitable variant caller for this region and purpose, but subsequent assembly with Flye resulted in fragmented assemblies which were incomplete for some samples. Further research is needed to evaluate the performance of various assemblers, phasing methods and variant callers within the SLA region specifically.

Separating the haplotypes is essential for identification of all SLA genes that an individual carries, and additionally enables direct inference of which SLA alleles are located on the same assembled haplotype. This provides a stronger basis for haplotype assignment in the absence of pedigree information than traditional typing approaches, where haplotypes are assigned through comparison to previously published haplotypes, (30, 34). However, this requires accurate phasing across the entire region spanning the SLA genes. The Class I region is particularly challenging because it contains substantial variation in copy number of SLA genes and pseudogenes (28), which can complicate phasing especially when copy number differs between the two haplotypes of an individual. For example, duplicated regions represented in the initial haploid assembly but present on only one haplotype may lack heterozygous variant calls and therefore provide little information for phasing. Reads originating from a haplotype containing fewer gene copies may also incorrectly align to duplicated copies represented in the assembly. Conversely, when duplicated gene copies are collapsed in the initial assembly, reads originating from multiple paralogous copies may map to a single assembled sequence, potentially generating spurious heterozygous variant calls. These issues represent an inherent limitation of our approach because phasing relies on read alignment and variant calling against an initial haploid assembly. This limitation was observed in six cases, where an alternative phase corresponded to previously described haplotypes, whereas the original phase resulted in two previously unobserved haplotypes. These samples also showed evidence of additional SLA gene or pseudogene copies, and regions lacking called variants, supporting the possibility that copy number variation contributed to local phase switches. These observations emphasize that haplotype assignments in structurally complex regions of the SLA locus should be interpreted with caution, particularly when copy number differs between haplotypes.

Although structural complexity in the SLA region can complicate phasing in specific cases, the majority of samples showed no signs of phasing issues. Therefore, the haplotype-resolved assemblies allowed the identification of candidate novel alleles, where the same sequence variants were consistently observed across animals within the same gene and haplotype. In this study, we identified three candidate novel *SLA-2* allele sequences that were not present in the IPD-MHC database. Two of these alleles were initially detected from the long-read haplotype-resolved assemblies, where multiple animals carried consistent sequence differences relative to the closest matching known *SLA-2* alleles. The third novel allele was carried by only a single long-read sequenced animal, which was too little evidence to consider it a potential novel allele initially. The presence of these candidate novel alleles was subsequently confirmed through targeted NGS-based SLA typing. Two of these novel alleles will require further sequencing to confirm the complete exon 2 sequence, as this sequence was not fully captured by the targeted sequencing approach used. Follow-up research will focus on performing all additional sequencing required to have the novel alleles fully validated for submission to the IPD-MHC database. Notably, the novel allele sequences obtained through our long-read based method were fully concordant with the targeted sequencing results. Together, these results demonstrate that haplotype-resolved long-read sequence-based assemblies provide a powerful approach for discovering candidate novel SLA alleles, while targeted sequencing provides and important independent means of validating candidate sequencing prior to formal allele assignment.

Furthermore, we observed a strong linkage between the assembly-derived SLA haplotypes and medium-density genotypes within the SLA region, allowing us to link SLA haplotypes to distinct SNP-based haplotypes in this population. By verifying these SNP-based SLA haplotype inferences using an independent dataset of targeted NGS-based SLA typing results, we show that SNP haplotypes have the potential to be an efficient and reliable method for predicting SLA haplotypes. This concept has previously been demonstrated (40) with a higher-density SNP panel, where fully unique co-occurrence of SNP haplotypes with PCR-SSP-based low-resolution SLA haplotypes was observed. However, the portability of such SNP panels across populations may be limited by differences in haplotype diversity, allele frequencies, and historical recombination events, which can alter the linkage disequilibrium between the selected SNPs and SLA haplotypes. Consequently, SNPs that accurately predict haplotypes in one population may not retain the same predictive power in genetically distinct populations. The SNP panel developed in the present study has been validated within the genetic backgrounds represented by the discovery population, and its predictive performance should therefore not be assumed to extend to genetically divergent populations. Therefore, validation in additional populations, including genetically diverse and indigenous pig populations, will be necessary to determine whether associations between SNPs and SLA haplotypes are maintained across different genetic backgrounds or whether alternative marker combinations provide improved predictive power. Such validation could ultimately facilitate the development of population-specific SNP panels optimized for accurate SLA haplotype prediction. SNP haplotypes may also be informative when used alongside other typing methods, as they provide insight into genetic relatedness among animals. In our NGS-typed dataset, SNP haplotypes facilitated the identification of a novel allele, as specific SNP haplotypes were consistently associated with the absence of alleles at the *SLA-2* loci.

In this study we demonstrate that high-quality genome assemblies constructed from long-read WGS data can provide valuable insight into SLA diversity. We do not propose this approach as a replacement for established SLA typing methods, which remain essential for precise allele typing and validation. Rather, we aim to highlight how the increasing availability of long-read WGS data across diverse pig populations can complement existing SLA research. As more high-quality long-read datasets become available, they have the potential to facilitate more comprehensive cataloguing of SLA alleles and haplotypes, including the identification of rare and putative novel variants that are currently absent from the IPD-MHC database. Furthermore, our results suggest that SLA haplotypes can be predicted from medium-density genotype data, although further validation across diverse genetic backgrounds will be required to assess the broader applicability of this approach. If validated, such SNP-based methods could provide a more accessible and cost-effective means of monitoring SLA diversity on population-wide scales, which can help us in ensuring sufficient genetic diversity to maintain healthy and resilient populations.

## Data Availability

The data presented in the study are deposited in the 4TU repository, DOI https://doi.org/10.4121/65ac5fae-a089-4e18-a438-6269cfa307ce.
