# Supplementary material for: Haplotype-resolved swine leukocyte antigen (SLA) gene typing using long-read whole genome sequencing
Source: Front Immunol. 2026 Oct 16;17:1903706. doi: 10.3389/fimmu.2026.1903706 (PMC13719319; doi:10.3389/fimmu.2026.1903706)
Supplement: Supplementary file 1 [file DataSheet1.docx]

***Supplementary Material***

Table S1: Positions of SNPs used to construct SNP haplotypes for Class I and Class II, based on Sscrofa 11.1.

| Chromosome | Class I | Class II |
| --- | --- | --- |
| 7 | 22608587 | 24829227 |
| 7 | 22616122 | 24865378 |
| 7 | 22727959 | 24979336 |
| 7 | 22758480 | 25049757 |
| 7 | 22767262 | 25082300 |
| 7 | 22805397 | 25091206 |
| 7 | 22805398 | 25138558 |
| 7 | 22852364 | 25176925 |
| 7 | 22951243 | 25205405 |
| 7 | 22975303 |  |
| 7 | 23037874 |  |
| 7 | 23143100 |  |
| 7 | 23153002 |  |
| 7 | 23174236 |  |
| 7 | 23266321 |  |
| 7 | 23360692 |  |
| 7 | 23406049 |  |
| 7 | 23412544 |  |
| 7 | 23426839 |  |
| 7 | 23481690 |  |
| 7 | 23504078 |  |
| 7 | 23515785 |  |
| 7 | 23515817 |  |

Table S2: SLA Class I haplotypes derived from the haplotype resolved assemblies where phase switches are suspected, and their corresponding reconstructed haplotypes.

| **Sample** | **Original Haplotype 1** | **Original Haplotype 2** | **Reconstructed Haplotype 1** | **Reconstructed Haplotype 2** |
| --- | --- | --- | --- | --- |
| 1 | 04:01 / 07:01 / NA | 08:17 / 04:01 / 04:02 | 04:01 / 04:01 / 04:02 (Hp-4b) | 08:17 / 07:01 / NA (unresolved) |
| 2 | 07:02 / 03:03 / 15:01 | 11:02 / 04:02 / 02:02 | 07:02 / 04:02 / 02:02 (Hp-32) | 11:02 / 03:03 / 15:01 (Hp-56) |
| 3 | 15:03 + 09:01 / 07:01 / 05:03 | 15:01 / 03:02 / 07:Novel | 15:01 + 09:01 / 07:01 / 05:03 (Hp-28) | 15:03 / 03:02 / 07:Novel (New^1^) |
| 4 | 15:01 / 03:02 / 07:01 | 11:01 / 04:02 / 02:02 | 15:01 / 04:02 / 02:02 (Hp-73) | 11:01 / 03:02 / 07:01 (Hp-25) |
| 5 | 14:02 / 03:02 / 07:01 | 11:01 / 04:03 / 11:04 | 14:02 / 04:03 / 11:04 (Hp-55) | 11:01 / 03:02 / 07:01 (Hp-25) |
| 6 | 15:01 / 11:04 / 04:03 | 14:02 + 09:01 / 07:01 / 05:03 | 15:01 + 09:01 / 07:01 / 05:03 (Hp-28) | 14:02 / 11:04 / 04:03 (Hp-55) |

Table S3: SLA Class I and II combined haplotypes (Hp) identified in Duroc, Synthetic (SY), Large White (LW), Landrace (LR), and Landrace x Large White crossbreds (LRxLW).

| **Hp** | ***SLA-1*** | ***SLA-2*** | ***SLA-3*** | ***SLA-DRB1*** | ***SLA-DQB1*** | **Count** | **Duroc** | **SY** | **LW** | **LR** | **LRxLW** |
| --- | --- | --- | --- | --- | --- | --- | --- | --- | --- | --- | --- |
| 1.1 | 01:01 | 01:01 | 01:01 | 01:01 | 01:01 | 6 |  |  | 5 |  | 1 |
| 1.12b | 01:01 | 01:01 | 01:01 | 06:01 | 07:01 | 1 |  |  |  |  | 1 |
| 1.14 | 01:01 | 01:01 | 01:01 | 09:01 | 08:01 | 1 |  |  | 1 |  |  |
| 1.27 | 01:01 | 01:01 | 01:01 | 09:01 | 09:01 | 3 |  |  | 3 |  |  |
| 4a.1 | 04:01:01 | 04:01 | 04:01 | 01:01 | 01:01 | 4 |  |  | 4 |  |  |
| 4a.14 | 04:01:01 | 04:01 | 04:01 | 09:01 | 08:01 | 4 |  | 3 |  |  | 1 |
| 4a.2 | 04:01:01 | 04:01 | 04:01 | 02:01 | 02:01 | 2 | 2 |  |  |  |  |
| 4a.26 | 04:01:01 | 04:01 | 04:01 | 11:02 | 04:03 | 1 |  |  | 1 |  |  |
| 4b.11 | 04:01:01 | 04:02:01 | 04:01 | 09:01 | 04:02 | 2 | 2 |  |  |  |  |
| 4b.2 | 04:01:01 | 04:02:01 | 04:01 | 02:01 | 02:01 | 4 | 4 |  |  |  |  |
| 4b.5 | 04:01:01 | 04:02:01 | 04:01 | 05:01 | 02:01 | 2 | 2 |  |  |  |  |
| 5.1 | 04:02 | 08:01 | 05:03 | 01:01 | 01:01 | 2 |  |  | 1 |  | 1 |
| 32.10 | 07:02 | 02:02 | 04:03 | 04:01 | 08:01 | 1 | 1 |  |  |  |  |
| 32.12a | 07:02 | 02:02 | 04:03 | 06:02 | 07:01 | 1 |  |  |  | 1 |  |
| 32.12b | 07:02 | 02:02 | 04:03 | 06:01 | 07:01 | 2 |  |  |  | 1 | 1 |
| 32.14 | 07:02 | 02:02 | 04:03 | 09:01 | 08:01 | 1 | 1 |  |  |  |  |
| 32.2 | 07:02 | 02:02 | 04:03 | 02:01 | 02:01 | 5 | 5 |  |  |  |  |
| 32.23 | 07:02 | 02:02 | 04:03 | 10:01 | 06:01 | 1 |  |  |  |  | 1 |
| 32.6 | 07:02 | 02:02 | 04:03 | 05:01 | 08:01 | 1 |  |  |  | 1 |  |
| New^4^.2 | 07:02 | 04:01 | 04:01 | 02:01 | 02:01 | 1 | 1 |  |  |  |  |
| New^2^.26 | 07:04 | 06:Novel | 05:07 | 11:02 | 04:03 | 2 |  |  | 1 |  | 1 |
| New^2^.27 | 07:04 | 06:Novel | 05:07 | 09:01 | 09:01 | 1 |  |  | 1 |  |  |
| New^2^.6 | 07:04 | 06:Novel | 05:07 | 05:01 | 08:01 | 1 |  |  |  |  | 1 |
| New^2^.NA | 07:04 | 06:Novel | 05:07 | 05:02 | 06:01 | 1 |  |  | 1 |  |  |
| 7.12b | 08:01 | 05:02 | 07:01 | 06:01 | 07:01 | 1 |  |  |  |  | 1 |
| New^6^.12b | 08:01 | 02:02 | 04:03 | 06:01 | 07:01 | 1 |  |  |  | 1 |  |
| New^6^.4 | 08:01 | 02:02 | 04:03 | 02:01 | 04:01 | 1 |  |  |  |  | 1 |
| New^6^.6 | 08:01 | 02:02 | 04:03 | 05:01 | 08:01 | 1 |  |  |  |  | 1 |
| 26a.20 | 08:08 | 10:05 | 05:02 | 06:02 | 03:03 | 1 |  | 1 |  |  |  |
| 26b.23 | 08:11 | 10:06 | 05:02 | 10:01 | 06:01 | 2 |  |  |  | 1 | 1 |
| 25.12b | 11:01 | 07:01 | 03:02 | 06:01 | 07:01 | 1 |  |  |  | 1 |  |
| 25.25 | 11:01 | 07:01 | 03:02 | 13:01 | 09:01 | 4 |  |  |  | 2 | 2 |
| 25.6 | 11:01 | 07:01 | 03:02 | 05:01 | 08:01 | 1 |  |  |  | 1 |  |
| 25.NA | 11:01 | 07:01 | 03:02 | 05:02 | 06:01 | 1 |  |  |  |  | 1 |
| 56.30 | 11:02 | 15:01 | 03:03 | 11:01 | 05:03 | 3 | 3 |  |  |  |  |
| 43.14 | 11:04 | 04:02:01 | 04:01 | 09:01 | 08:01 | 1 | 1 |  |  |  |  |
| 43.2 | 11:04 | 04:02:01 | 04:01 | 02:01 | 02:01 | 1 | 1 |  |  |  |  |
| 35.23 | 12:01+13:01 | 10:01 | 05:02 | 10:01 | 06:01 | 1 |  |  | 1 |  |  |
| New5.12a | 12:01+13:01 | 06:02 | 04:04 | 06:02 | 07:01 | 1 |  |  |  | 1 |  |
| 62.12a | 14:01 | 06:02 | 04:04 | 06:02 | 07:01 | 1 |  |  |  | 1 |  |
| 55.12a | 14:02 | 11:04 | 04:03 | 06:02 | 07:01 | 2 |  |  |  | 2 |  |
| 55.12b | 14:02 | 11:04 | 04:03 | 06:01 | 07:01 | 5 |  |  | 3 | 2 |  |
| 55.14 | 14:02 | 11:04 | 04:03 | 09:01 | 08:01 | 1 |  |  |  |  | 1 |
| 55.15b | 14:02 | 11:04 | 04:03 | 04:02 | 02:02 | 4 |  | 4 |  |  |  |
| 55.25 | 14:02 | 11:04 | 04:03 | 13:01 | 09:01 | 1 |  |  |  | 1 |  |
| 55.26 | 14:02 | 11:04 | 04:03 | 11:02 | 04:03 | 1 |  |  |  |  | 1 |
| 55.4 | 14:02 | 11:04 | 04:03 | 02:01 | 04:01 | 2 |  |  | 2 |  |  |
| 55.6 | 14:02 | 11:04 | 04:03 | 05:01 | 08:01 | 9 |  |  |  | 9 |  |
| 73.12b | 15:01 | 02:02 | 04:03 | 06:01 | 07:01 | 2 |  |  |  | 2 |  |
| 28.23 | 15:01+09:01 | 05:03 | 07:01 | 10:01 | 06:01 | 4 |  |  |  | 2 | 2 |
| New1.41 | 15:03 | 07:Novel | 03:02 | 04:01 | 05:01 | 3 |  |  |  | 2 | 1 |
| New3.14 | 21:01+11:03 | 16:03 | 05:03 | 09:01 | 08:01 | 1 |  |  |  |  | 1 |
| New3.6 | 21:01+11:03 | 16:03 | 05:03 | 05:01 | 08:01 | 1 |  |  |  |  | 1 |

**(A)**

**(B)**

Figure S1: **(A)** Self-alignment dot-plot and **(B)** self-mapped read coverage + heterozygous variant density across the region holding the classical SLA Class I genes of sample 1

**(A)**

**(B)**

Figure S2: : **(A)** Self-alignment dot-plot and **(B)** self-mapped read coverage + heterozygous variant density across the region holding the classical SLA Class I genes of sample 2

**(A)**

**(B)**

Figure S3: : **(A)** Self-alignment dot-plot and **(B)** self-mapped read coverage + heterozygous variant density across the region holding the classical SLA Class I genes of sample 4

**(A)**

**(B)**

Figure S4: **(A)** Self-alignment dot-plot and **(B)** self-mapped read coverage + heterozygous variant density across the region holding the classical SLA Class I genes of sample 5

**(A)**

**(B)**

Figure S5: : **(A)** Self-alignment dot-plot and **(B)** self-mapped read coverage + heterozygous variant density across the region holding the classical SLA Class I genes of sample 6

**(A)**

**(B)**

Figure S6: Linkage disequilibrium (R^2^>0.10) in Landrace between SNPs used to construct SNP haplotypes for **(A)** Class I and **(B)** Class II

**(A)**

**(B)**

Figure S7: Linkage disequilibrium (R^2^>0.10) in Large White between SNPs used to construct SNP haplotypes for **(A)** Class I and **(B)** Class II

**(A)**

**(B)**

Figure S8: Linkage disequilibrium (R^2^>0.10) in Duroc between SNPs used to construct SNP haplotypes for **(A)** Class I and **(B)** Class II

**(A)**

**(B)**

Figure S9: Linkage disequilibrium (R^2^>0.10) in Synthetic between SNPs used to construct SNP haplotypes for **(A)** Class I and **(B)** Class II
